# The effect of coenzyme Q10 on cryotolerance of *in vivo*-derived mouse embryos

**DOI:** 10.5935/1518-0557.20240029

**Published:** 2024

**Authors:** Parichehr Sadat Hosseini, Iraj Jafari Anarkooli, Alireza Abdanipour, Mitra Arianmanesh

**Affiliations:** 1 Department of Anatomical Sciences, School of Medicine, Zanjan University of Medical Sciences (ZUMS), Zanjan, Iran

**Keywords:** antioxidant, Coenzyme Q10, cryopreservation, mouse embryo

## Abstract

**Objective:**

Cryopreservation has some adverse effects on embryos including cell metabolism reduction, mitochondria and plasma membrane damage, excess production of 'Reactive Oxygen Species' and damage to DNA. In the present study. In this study we assessed the effect of coenzyme Q10 as an exogenous antioxidant on mouse embryos following cryopreservation.

**Methods:**

We collected mice embryos at the morula stage from uterine horns on the third day of gestation. The morulae were divided into 9 groups (1 control, 2 vehicles and 6 experimental), then vitrified. The culture and/or vitrification media of the experimental groups were supplemented by 10 or 30 µM of CoQ10. After one week, the embryos were warmed and then cultured. After 48 hours of embryo culture, the blastocyst rate, total cell number, viability; and after 72 hours of embryo culture, we assessed the hatching rate.

**Results:**

Blastocyst rate and hatching rate were significantly reduced in the groups containing 30 µM CoQ10 supplemented culture media compared to other groups (*p*<0.05). The hatching rate in the groups containing 10 µM CoQ10 supplemented in both culture and vitrification media was significantly higher than in the other groups (*p*<0.05). In groups containing 10 µM CoQ10 supplemented culture media, the viability was higher than that in the other groups (*p*<0.05).

**Conclusions:**

It seems that CoQ10 in a dose-dependent manner is able to improve hatching rate and viability following cryopreservation through its antioxidant and anti-apoptotic properties, and through the production of ATP.

## INTRODUCTION

Embryo cryopreservation is a technique enabling fertility to be preserved. It is usually applied in patients with reproductive and non-reproductive cancers, women with early menopause, and women who delay the age of motherhood (Al-Hasani *et al*., 2007). Also, in embryo cryopreservation the number of embryos transferred (ET) per cycle decreases and the number of cycles of ovulation induction reduce with subsequent risk of complications, for instance, ovarian hyperstimulation syndrome (Batuhan & Safaa, 2010). Indeed, in the majority of fertility clinics, all embryos are first frozen, then thawed and transferred to the uterus, when the endometrium is under receptive conditions and is free from the effects of hormonal drugs utilized in ovarian stimulation (Konc *et al*., 2014).

Despite all advances in cryopreservation techniques, cryopreservation can still result in various damages to the cell and disturb the cell metabolism. Cryopreservation induces apoptosis and necrosis through biochemical changes, a decrease in energy levels, activation of caspases, ionic imbalance, aggregation of free radicals, damage to plasma membrane, changes in osmotic pressure, and chromosomal deviation (Tas *et al*., 2023; Baust *et al*., 2009; Kopeika *et al*., 2014). Also, some cryoprotectants used in cryopreservation may increase embryo DNA fragmentation (Rajaei *et al*., 2005) leading to reduced embryo viability (Park *et al*., 2006; Kader *et al*., 2010). *In vitro* conditions increase the glycolysis pathway while it decreases the oxidation-phosphorylation pathway. As a consequence, it causes an imbalance in oxidation-reduction processes, which negatively affects the function of mitochondria (Abe *et al*., 2002; Sudano *et al*., 2013). Also, the excessive production of reactive oxygen species (ROS) during cryopreservation causes direct damage to the mitochondria, leading to activation of proteases and caspases, DNA fragmentation, apoptosis, and impairment in cell division and embryonic development (Velez-Pardo *et al*., 2007). ROS ultimately cause oxidative stress, which is one of the deleterious impacts of cryopreservation (Chatterjee & Gagnon, 2001; Wang *et al*., 2003). Oxidative stress induces membrane lipid peroxidation, oxidation of amino acids and nucleic acids, apoptosis and necrosis, which may reduce the viability of embryos produced by *in vitro* fertilization (Johnson & Nasr-Esfahani, 1994; Ali *et al*., 2003; Kitagawa *et al*., 2004). To overcome the detrimental impacts of ROS on embryos, exogenous antioxidants have frequently been utilized. Studies have demonstrated that the addition of antioxidants to the culture and vitrification media of *in vitro* produced embryos increases the antioxidant capacity of embryos to scavenge ROS, with subsequent improved embryo viability (Rocha *et al*., 2012; Castillo-Martín *et al*., 2014).

Coenzyme Q10 (CoQ10) is an electron carrier in the mitochondrial electron respiratory chain of most eukaryotic cells (Yang *et al*., 2021). CoQ10 has been implicated in many various functions in the cell, including antioxidant, inhibition of protein and DNA oxidation, inhibition of lipid peroxidation (Gutierrez-Mariscal *et al*., 2020), reduction of apoptosis induced by oxidative stress (Gollapudi & Gupta, 2016) and DNA replication and repair (Brown & McCarthy, 2023). Embryo studies have indicated that CoQ10 prevents DNA fragmentation (Talevi *et al*., 2013; Gualtieri *et al*., 2014), increases total antioxidant capacity (TAC) (Kashka *et al.*, 2015), enhances enzymatic antioxidant activity (Nadjarzadeh *et al.*, 2014; Hosseinzadeh *et al*., 2017), and protects embryos from oxidative damages (Özcan *et al*., 2016; Liang *et al*., 2017). CoQ10 also improves membrane stability and ionic balance (Stojkovic *et al*., 1999; El Refaeey *et al*., 2014). CoQ10 is able to reduce bovine oocyte apoptosis during in vitro maturation (IVM), in particular, after oocyte cryopreservation (Ruiz-Conca *et al*., 2022) it increases human oocyte maturation rates during IVM, and it decreases post-meiotic aneuploidies in older women (Ma *et al*., 2020; Rodríguez-Varela & Labarta, 2021; Santos *et al*., 2006). CoQ10 acts not only in the mitochondrial respiratory chain and in various aspects of cell metabolism, but it also acts as a primary ROS scavenger. In addition, since reduction of energy levels and damage to mitochondria is one of the outcomes of embryo cryopreservation (Kopeika *et al*., 2014), it is likely that CoQ10 has the potential ability to improve mitochondrial energy production (Meldrum *et al*., 2016).

Due to the role of CoQ10 in cell growth and energy production (ATP synthesis), its protective effects against oxidative stress, and since there is no reliable and complete study showing the effects of this antioxidant on the protection of mouse embryos against ROS, it is assumed that CoQ10 may be a good candidate to protect the embryos against the detrimental impacts of cryopreservation. In this study, CoQ10, as a mitochondrial energy enhancer, was added to the culture and vitrification media to evaluate developmental competence, cryotolerance, and the viability rate of mice embryos following cryopreservation.

## Materials and methods

### Substances

All chemicals were obtained from Sigma Aldrich Co. (St. Louis, Missouri, United States) unless otherwise stated.

### Animal care and collection of mice embryos

All procedures relating to the care and use of animals were approved by the Ethics Committee of the Zanjan University of Medical Sciences. Male and female NMRI mice aged 8-10 weeks were kept at 25ºC, 40-60% relative humidity, 12 hours of light and 12 hours of darkness with free access to water and food at the animal lab.

7.5IU PMSG (Pregnant Mare Serum Gonadotropin) (Folligon^®^, Intervet, Netherlands, HOR-272) were injected intraperitoneally (IP) into each female mouse (a total of 32) at noon (12:00). After 48h, 7.5IU hCG (Human Chorionic Gonadotropin) (Bioscience, GmbH, Germany) were administered through IP injection, then each female mouse was placed in a male mouse cage. The next morning, the female mice were examined for vaginal plaque, indicating sexual intercourse had taken place. The mice with vaginal plaque were considered pregnant (day 1). On the third day of gestation, the mice were sacrificed by cervical dislocation and the uterine horns were removed from both sides and transferred to petri dishes containing large droplets of T6 medium (473 mg NaCl, 100 mg KCl, 5 mg NaH_2_PO_4_, 10 mg MgCl_2_,6H_2_O, 26mg CaCl_2_,6H_2_O, 210 mg NaHCO_3_, 200 µL Na-lactate 100%, 3mg Na-pyruvate, 100 mg glucose, 6 mg penicillin, 5 mg streptomycin, 1 mg phenol red and 0.6mg EDTA to make 100mL T6 medium) plus 4% BSA (Bovine Serum Albumin) (Sigma Aldrich Co., A3311). The T6 medium supplemented with 4% BSA was then flushed into the uterine horns using an insulin syringe. The embryos released into the culture medium were evaluated by a stereomicroscope (Motic^®^, SMZ-168, China) and then the embryos at the morula stage and with good morphological quality were collected and transferred with 50 µL/drop of T6 medium supplemented with 4% BSA and overlaid with mineral oil. The embryos were then incubated in 5% CO_2_ incubator (New Brunswick™, Galaxy 170 S, USA) at 37ºC prior to vitrification.

### Embryo vitrification

The morulae were initially incubated in the equilibration solution (ES) of 7.5% ethylene glycol (EG) (Sigma Aldrich Co., 324558) + 7.5% dimethyl sulfoxide (Me_2_SO) (Sigma Aldrich Co., D2650) in T6 medium supplemented with 20% human serum albumin (HSA) for up to 7 min at room temperature. Equilibrated embryos were then exposed to the vitrification solution (VS) of 15%EG + 15%Me_2_SO + 0.5M sucrose in T6 medium supplemented with 20% HSA for 1 min at room temperature then loaded onto the tips of the cryotops (KITAZATO^®^, Japan) and immediately plunged into the liquid nitrogen and stored for a period of one week.

For warming, vitrified embryos were removed from the liquid nitrogen and were quickly introduced into a large droplet of the first warming solution (WS1) containing T6 + 20% HSA (Biotest, UK) + 1 M sucrose for 1 min on a warm stage at 37°C. The embryos were then transferred to a second warming solution (WS2) containing T6 + 20%HSA + 0.5M sucrose and incubated for 3 min at room temperature. In the next step, the embryos were transferred to the third warming solution (WS3) containing T6 + 20%HSA + 0.25M sucrose and incubated for 3min at room temperature. The embryos were then washed in 20 droplets containing the basic medium (T6 + 20% HSA) and then transferred to the culture medium containing T6 + with or without CoQ10/ethanol supplemented by 4% BSA and incubated in 5% CO_2_ incubator at 37 ºC. Finally, approximately half of the frozen-thawed embryos (7 embryos) were assessed 48 h post-warming to determine the percentages of blastocyst formation rate, total cell number (TCN), and viability and the remaining half (6 embryos) were assessed 72 h post-warming to determine the percentage of hatched blastocysts.

### Experimental design

**Figure 1:**
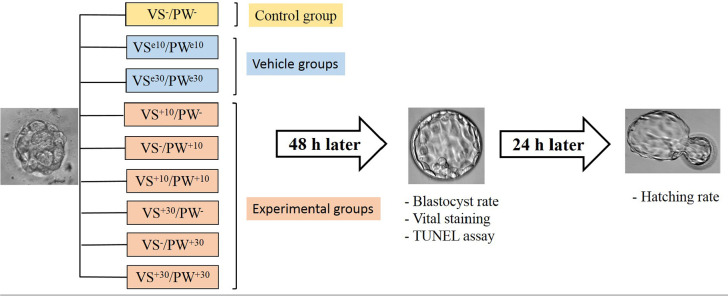
A diagram illustrating the experimental design. In vivo-produced morulae were randomly divided into 9 groups: 1 control (VS-/PW-) in which nothing was added to the vitrification solution and the culture medium; 2 vehicles (VS^e10^/PW^e10^ and VS^e30^/PW^e30^) in which both vitrification solution and culture medium were supplemented by 10 ul/ml or 30 ul/ml of ethanol, and 6 experimental groups (VS^+10^/PW^+10^, VS^+30^/PW^+30^, VS^+10^/PW^-^, VS^+30^/PW^-^, VS^-^/PW^+10^, VS^-^/PW^+30^), in which vitrification solution and/or culture medium were supplemented by 10 µM or 30 µM of CoQ10. VS: vitrification solution, PW: post warming, e: ethanol.

As demonstrated in Figure 1, the collected morulae were randomly assigned to experimental and control groups (13 embryos/group). In two experimental groups, both culture and vitrification media were supplemented with two different doses (10 and 30 µM) of CoQ10 (Abcam, Cambridge, United Kingdom) (VS^+10^/PW^+10^ and VS^+30^/PW^+30^) (VS: vitrification solution, PW: post warming medium). In two other experimental groups, only vitrification medium was supplemented by two different doses (10 and 30 µM) of CoQ10 (VS^+10^/PW^-^ and VS^+30^/PW^-^). In two other experimental groups, only culture medium was supplemented by two different doses (10 and 30µM) of CoQ10 (VS^-^/PW^+10^ and VS^-^/PW^+30^). The culture and vitrification of vehicle groups were supplemented with two different doses of ethanol (10 & 30µL/mL) CoQ10 solvent (VS^e10^/PW^e10^ and VS^e30^/PW^e30^). Nothing was added into the culture and vitrification media of the control group (VS^-^/PW^-^). All experiments for each group were carried out in two replicates.

### Embryo developmental competence assessment

**Figure 2:**
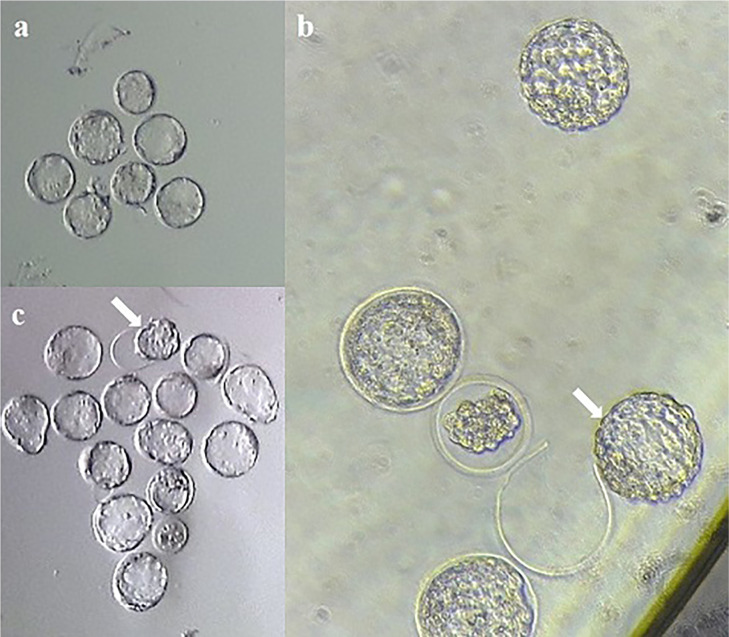
The post-warming embryos at blastocyst stage. A. Blastocysts at 48 h after warming, magnification: ×50. B. Blastocysts at 72 h after warming, magnification: ×50. An arrow indicates a blastocyst which is hatching from zona pellucida C. Blastocysts at 72 h after warming, magnification: ×200. An arrow indicates a blastocyst which hatched from the zona pellucida.

Forty-eight hours after warming, to assess blastocyst rate, the morphology of embryos was assessed under a stereomicroscope and the ratio of the number of blastocysts to the total number of embryos was recorded in percentages (Figure 2). To assess blastocyst morphology, we used a grading system based on the degree of cavity (Du *et al*., 2016). There are six groups in this grading system: a blastocyst with blastocoel which was less than 50% of blastocyst size was classified as group 1; early blastocyst with blastocoel was greater than 50% of blastocyst size, classified as group 2; full blastocyst with blastocoel which completely fills the blastocyst was classified as group 3; expanded blastocyst was classified as group 4, hatching blastocyst was classified as group 5; and hatched blastocyst was classified as group 6 (Du *et al*., 2016). 72h after warming, to assess hatching rate, the number of blastocysts which were categorized into stages 5 and 6 was calculated and the ratio of the number of hatched blastocysts to the total number of blastocysts was recorded in a percentage (Figure 2).

### Vital staining to detect viable and dead blastomeres and total cell number (TCN)

Forty-eight hours after warming, the blastocysts in stages 3 and 4 were randomly selected for vital staining. To make a color solution, 300 µg propidium iodide (Sigma Aldrich Co., P4170) and 20 µg bisbenzemide (Hoechst 33258) (Sigma Aldrich Co., B1155) were added to 1 mL of T6 medium supplemented by 4% BSA, then it was placed in an incubator (37°C and 5% CO_2_). Blastocysts were first washed in PBS at 37°C, then transferred to droplets containing pre-prepared color solution, and placed in an incubator for 30 min. Afterwards, the embryos were washed again at 37°C in PBS and then fixed in 2.5% glutaraldehyde (Merck, Darmstadt, Germany) solution for 5 min at room temperature. Then, the blastocysts were washed and transferred to a slide with a drop of 10% glycerol and the coverslip. Evaluation was performed using a fluorescence microscope (Olympus, BX51, Tokyo, Japan) with a 450-500 nm filter. In this method, dead cells absorb both Hoechst and propidium iodide and thus exhibit more radiation than live cells. In each blastocyst, 5 fields were evaluated and the total number of blastomeres as well as the ratio of live blastomeres to the total number of blastomeres were recorded in percentages.

### Statistical analysis

Normal distribution of data was analyzed by histogram and statistical tests using Shapiro-Wilk and Kolmogorov-Smirnov tests with the SPSS^®^ software (Version 16, IBM, Chicago, USA). To assess viability and DFI, normally distributed data were subjected to one way ANOVA and Bonferroni post hoc tests to assess the significance of differences. Statistical comparisons between specific groups were carried out using the student’s T-test. Chi-square test and then multiple t-tests were used to assess TCN, blastocyst rate and hatching rate. Differences were considered to be significant if *p*<0.05.

## RESULTS

### Effect of Q10 on embryo developmental competence

The blastocyst rate in VS^-^/PW^+30^ and VS^+30^/PW^+30^ groups was significantly lower than in the control, vehicle and other experimental groups (*p*<0.05); but these two groups did not differ significantly. Any significant difference was not observed between other groups (Table 1).

**Table 1:** The comparison of blastocyst rate, hatching rate, and total cell number in study groups.

Groups	Number of Morulae	Blastocyst Rate (%)	Hatching Rate (%)	TCN
**VS^-^/PW^-^**	9	93.75 ^ab^	28.57 ^abcd^	67.00
**VS^e10^/PW^e10^**	8	92.86 ^ab^	20.00 ^abce^	61.00
**VS^e30^/PW^e30^**	7	84.52 ^ab^	20.00 ^abce^	58.00
**VS^+10^/PW^-^**	9	88.75 ^ab^	50.00 ^acde^	63.00
**VS^-^/PW^+10^**	9	95.00 ^ab^	40.00 ^acde^	66.00
**VS^+10^/PW^+10^**	8	95.00 ^ab^	66.67^bcde^	63.67
**VS^+30^/PW^-^**	7	78.89 ^ab^	28.57 ^abcd^	68.5
**VS^-^/PW^+30^**	8	28.89	0.01 ^abde^	54.5
**VS^+30^/PW^+30^**	8	23.21	16.67 ^abce^	52.00

All data is drawn from two replicates. Data are reported as Mean. ^abcde^: significant difference between groups (*p*≤0.05). In the comparison of blastocyst rate: a vs VS-/PW^+30^, b vs VS^+30^/PW^+30^. In the comparison of hatching rate: ^a^ vs VS^+10^/PW^+10^, b vs VS^-^/PW^+10^ and VS^+10^/PW^-^, c vs VS^-^/PW^+30^, d vs VS^+30^/PW^+30^, VS^e10^/PW^e10^, and VS^e30^/PW^e30^ and e vs VS^-^/PW^-^ and VS^+30^/PW-. In the comparison of TCN, there was no significant difference between the studied groups. VS: Vitrification Solution, PW: Post Warming, ^e10^: presence of 10 µL/ml ethanol, ^e30^: presence of 30 µL/ml ethanol, ^+10^: presence of 10 µM CoQ10, ^+30^: presence of 30 µM CoQ10, ^-^: absence of CoQ10.

The rate of hatching blastocysts in the VS^+10^/PW^+10^ group was significantly higher than in the control, vehicle and other experimental groups (*p*<0.05); whereas in VS^-^/PW^+30^ group was significantly lower than in the control, vehicle and other experimental groups (*p*<0.05) (Table 1). The hatching rate was significantly lower in the VS^+30^/PW^+30^, VS^e10^/PW^e10^, and VS^e30^/PW^e30^ groups compared to the control and other experimental groups (*p*<0.05), excluding VS^-^/PW^+30^ group (Table 1). Also, there was a significant increase in the hatching rate in VS^-^/PW^-^ and VS^+30^/PW^-^ groups, in comparison with the VS^-^/PW^+30^, VS^+30^/PW^+30^, VS^e10^/PW^e10^, and VS^e30^/PW^e30^ groups; however, there was a significant decrease compared to the VS^-^/PW^+10^, VS^+10^/PW^-^, and VS^+10^/PW^+10^ groups (*p*<0.05) (Table 1).

### Effect of Q10 on viability of blastomeres and TCN

**Figure 3:**
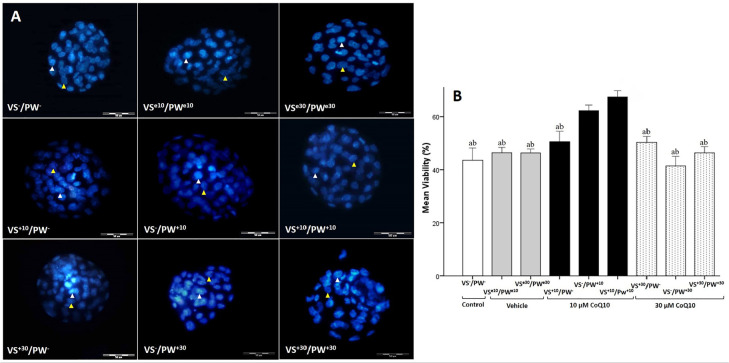
Assessment of blastocysts viability using vital staining. A. Representative image of blastocysts from the study groups. The blastocysts were stained by propidium iodide and Hoechst 33358, (dead blastomeres appear in light blue and shown with white arrowheads. Alive blastomeres shown with yellow arrowheads: magnification: ×400, scale bars: 50 µm). B. Comparison of mean viability in the study groups. All data is drawn from two replicates. Data are reported as Mean±SD. ^ab^: significant difference between groups (*p*≤0.05).^a^ vs VS^+10^/PW^+10^, ^b^ vs VS^-^/PW^+10^. VS: Vitrification Solution, PW: Post Warming, e10: presence of 10 µL/ml ethanol, ^e30^: presence of 30 µL/ml ethanol, ^+10^: presence of 10 µM CoQ10, ^+30^: presence of 30 µM CoQ10, ^-^: absence of CoQ10.

The rate of viability was significantly higher in the VS^-^/PW^+10^ group (62.21±1.06) compared to the VS^-^/PW^-^ (43.58±2.29), VS^e10^/PW^e10^ (46.40±0.98), VS^e30^/PW^e30^ (46.33±0.74), VS^+10^/PW^-^ (50.56±1.95), VS^+30^/PW^-^ (50.28±1.16), VS^-^/PW^+30^ (41.43±1.83), and VS^+30^/PW^+30^ (46.38±1.16) (*p*<0.05) groups (Figure 3). Also, the rate of viability was significantly higher in the VS^+10^/PW^+10^ group (67.40±1.23) compared to the VS^-^/PW^-^ (43.58±2.29), VS^e10^/PW^e10^ (46.40±0.98), VS^e30^/PW^e30^ (46.33±0.74), VS^+10^/PW^-^ (50.56±1.95), VS^+30^/PW^-^ (50.28±1.16), VS^-^/PW^+30^ (41.43±1.83), and VS^+30^/PW^+30^ (46.38±1.16) (*p*<0.05) groups (Figure 3).The VS^-^/PW^+30^ group (41.43±1.83) had a mean viability lower than those from the other groups; but it was only significant when compared to the VS^-^/PW^+10^ (62.21±1.06) and VS^+10^/ PW^+10^ (67.40±1.23) groups (*p*<0.05) (Figure 3). The analysis of total cell number in different groups showed no statistically significant difference between groups (Table 1).

## DISCUSSION

In this study, CoQ10 at a dose of 10 µM had positive effects on some of the embryonic developmental parameters following cryopreservation (including blastocyst rate, hatching rate, and viability). The results of our study also showed that the addition of coenzyme Q10 to the culture medium caused a significant change in the studied parameters and the presence of coenzyme Q10 in the vitrification medium did not show its effects, which is likely because in the vitrification method, the embryo is vitrified with a very small amount of vitrification medium and the effect of the vitrification medium in this method was negligible. Similar to our results, Liang *et al*. (2017) reported that CoQ10 increases cleavage, blastocyst rate, hatching rate and expression of hatching genes; while it decreases ROS, DNA damage and apoptosis in porcine embryos. CoQ10 plays an anti-apoptotic and antioxidant role by stabilizing the membrane, maintaining ionic balance in cells, and increasing the production of energy (Stojkovic *et al.*, 1999; Marriage *et al*., 2004; El Refaeey *et al*., 2014; Özcan *et al*., 2016; Liang *et al*., 2017).

**Figure 4:**
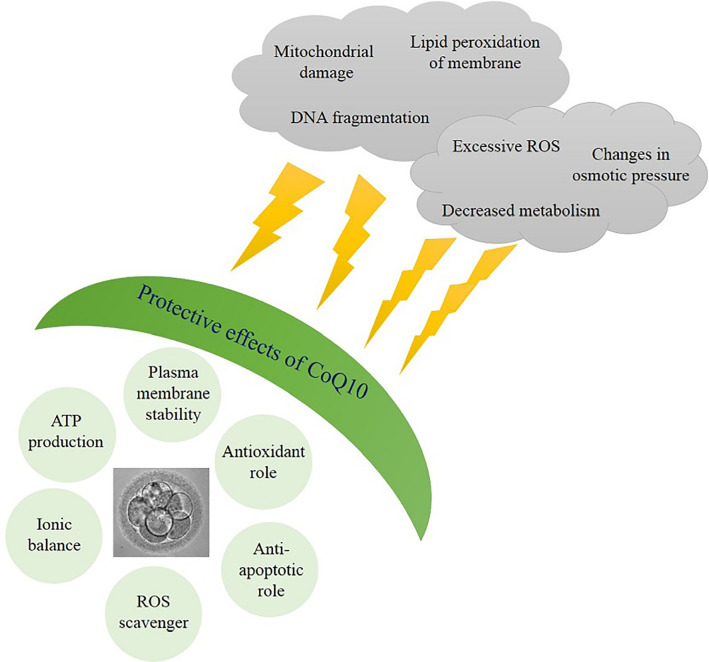
The protective effects of CoQ10 on embryo. A schematic image indicating protective effects of CoQ10 on embryo against detrimental impacts of *in vitro* conditions.

The ability of mitochondrial DNA replication from MII oocytes to blastocysts is lost, so the number of mitochondria per cell decreases (Van Blerkom, 2008). Defects in embryonic development are associated with mitochondrial defects, and inadequate mitochondrial storage can lead to impaired embryonic development (Ma *et al*., 2020). On the other hand, *in vitro*, there is an increase in the activity of the glycolysis pathway and inhibition of the oxidative phosphorylation pathway (Sudano *et al*., 2013). The number and distribution of mitochondria are also affected by vitrification/warming (Zhao *et al*., 2011). Considering the above, we can say that improving mitochondrial energy production in cases of poor embryonic development can lead to improved results (Meldrum *et al*., 2016). Various studies have shown that coenzyme Q10 increases ATP production (El Refaeey *et al.*, 2014; Marriage *et al*., 2004). Therefore, CoQ10 acts as a shield to protect the embryo from induced stresses and damage caused by cryopreservation, including excessive ROS production and subsequent oxidative stress, DNA fragmentation, lipid peroxidation, decreased metabolism, mitochondria damages and alterations in osmotic pressure (Turunen *et al*., 2004; Littarru & Tiano, 2007; Gollapudi & Gupta, 2016) (Figure 4).

A study on bovine embryos conducted by Stojkovic *et al*. (1999) showed that the effect of CoQ10 on the embryo is dose dependent. They reported that the effective dose is 30 µM; whereas it is not effective at doses lower than 30 µM; and has no effect including adverse effect at doses above 30 µM (Stojkovic *et al*., 1999). Similarly, in the present study, CoQ10 at 10µM dose (low dose) showed positive effects on embryo developmental competence following cryopreservation; while CoQ10 at a 30µM dose (high dose) was found to be ineffective on TCN and viability; and had negative effects on the blastocyst and hatching rates. It may be suggested that the negative effects of CoQ10 at high doses may be the result of its effects at the molecular level, including changes in the gene expression involved in early embryonic development. Therefore, it seems that, this hypothesis should be explained in more details by further studies.

Most of the ROS in the cells are produced in the mitochondrial respiratory chain (Zhang *et al*., 2022). It has also been widely accepted that the accumulation of the reduced form of CoQ10 (ubiquinol) produces superoxide ions (a type of ROS) through a mechanism called retrograde electron transfer, a mechanism which stabilizes the ratio of ubiquinone to ubiquinol (Pryde & Hirst, 2011), implicating the reverse effects of CoQ10 at high doses.

Collectively, the findings of this study indicate that supplementation of culture medium with 10 µM CoQ10, as an exogenous antioxidant, improves hatching rate, increases embryo viability; and in general, it can be concluded that coenzyme Q10 has protective effects on mice embryo development following cryopreservation. Coenzyme Q10 appears to have these beneficial effects through its antioxidant and anti-apoptotic properties, and through the production of ATP. To determine the mechanisms of coenzyme Q10 functions as well as to explain adverse effects of CoQ10 at high doses, further studies are required to unravel the exact mechanisms at the molecular levels.
